# Pathogenic mechanisms of deregulated microRNA expression in thyroid carcinomas of follicular origin

**DOI:** 10.1186/1756-6614-4-S1-S1

**Published:** 2011-08-03

**Authors:** Juliane Braun, Stefan Hüttelmaier

**Affiliations:** 1 Institute of Molecular Medicine, Section for Molecular Cell Biology, Martin Luther University of Halle-Wittenberg, ZAMED Heinrich-Damerow-Str.1, 06120 Halle, Germany

## Abstract

Thyroid cancer is one of the most common malignancies of the endocrine system with increasing incidence. The vast majority of thyroid carcinomas derive from thyroid hormone producing follicular cells. Carcinomas of follicular origin are classified as follicular (FTCs), papillary (PTCs), partially differentiated (PDTCs) or anaplastic (ATCs) thyroid carcinomas. While FTCs and PTCs can be managed effectively, ATCs are considered one of the most lethal human cancers. Despite the identification of various genetic alterations, pathogenic mechanisms promoting the progression of thyroid carcinomas are still largely elusive. Over the recent years, aberrant microRNA expression was revealed in all as yet analyzed human cancers, including thyroid carcinomas. In view of the rapidly evolving perception that deregulated microRNA expression serves a pivotal role in tumor progression, microRNAs provide powerful tools for the diagnosis of thyroid carcinomas as well as the identification of potential therapeutic targets. Here, we summarize recent findings on microRNA signatures in thyroid carcinomas of follicular origin and discuss how deregulated microRNA expression could promote cancer progression.

## Introduction

Thyroid carcinomas represent the most common cancer of the endocrine system [1]. More than 95% of these carcinomas originate from follicular thyroid cells, whereas only 3% are of C-cell origin, referred to as medullary thyroid carcinomas (MTCs) [2]. The most frequent follicular tumors are benign hyperplastic adenomas (FTAs), whereas papillary thyroid carcinomas (PTCs) are the most frequent thyroid carcinoma (approximately 90%) [3]. PTCs are composed of well-differentiated epithelial cells and can be distinguished by visible changes in nuclear morphology and appearance [4]. Follicular thyroid carcinomas (FTCs) with a prevalence of less than 10% are morphological similar to FTAs but capable of vascular invasion [2]. Although some of these well-differentiated carcinomas behave aggressively, the vast majority of PTCs and FTCs can be managed effectively. In contrast, the rare (2-7%) undifferentiated, anaplastic thyroid carcinomas (ATCs) behave very aggressively, rapidly invade adjacent tissues and are thus considered one of the most lethal human cancers [2]. At present there is no effective treatment of ATCs and death usually occurs within six months after diagnosis [5]. ATCs are characterized by partially or completely undifferentiated cells with a high mitosis rate, necrotic areas, spindle-like cell morphologies as well as giant and occasionally squamous cells [5,6]. Poorly differentiated thyroid carcinomas (PDTCs) present an ‘intermediate’ entity. They appear partially de-differentiated compared to FTCs or PTCs and typically behave more aggressively [5]. Several of these tumors arise *de novo*, whereas others seem to originate from PTCs or FTCs [*7*].

Thyroid tumors are supposed to be mainly monoclonal malignancies arising based on somatic mutations of progenitor cells. Putative risk factors for genomic instabilities are radiation exposure, active oxygen-species (H_2_O_2_ is necessary for thyroid hormone synthesis) and estrogen. Historical events exhibit radiation exposure as the major risk for PTCs, since atomic bomb survivors and Chernobyl victims frequently developed these tumors [8]. One of the major risks for FTCs is dietary iodine deficiency resulting in thyroid proliferation (endemic goiter) as a compensatory mechanism [9]. Genetic predisposition associated thyroid cancers are known for familial polyposis coli (mutations in APC), Cowden disease (mutations in PTEN) and Werner syndrome (mutations in WRN) [2]. Moreover, two sequence variants of *loci* 9q22.33 and 14q13.3 were found to be associated with a higher risk of FTCs and PTCs [10].

Genetic alterations in oncogenes that are involved in the activation of cell signalling pathways have been observed in the vast majority of malignant thyroid carcinomas. Mutation of the BRAF gene is most prominent in PTCs with an appearance up to 50% [2,11,12]. One major gain-of-function mutation of BRAF, a substitution of valine to glutamate at position 600 (V600E), results in constitutive activation of the MAPK pathway [13,14]. RAS gene (KRAS, HRAS, NRAS) mutations instead were found in all thyroid cancers: FTAs (frequency: 20-40%), FTCs (frequency: 40-50%), PDTCs (frequency: 20-55%) and ATCs (frequency: 20-60%) [2,11,12]. Activation of the G-protein RAS stimulates MAPK and other signalling pathways like PI3K/AKT. Less frequent are RET rearrangements (20-40% in PTCs, ~10% in PDTCs) due to inter-chromosomal translocations. In ATCs and PDTCs, CTNNB1 (0-25% in PDTCs, 66% in ATCs) and TP53 mutations (20-38% in PDTCs; 67-88% in ATCs) were observed [2,11,12,15,16]. NTRK1 rearrangements were exclusively identified in PTCs (~10%). The proto-oncogene NTRK1 (also known as TRK) encodes a trans-membrane tyrosine-kinase receptor for nerve growth factor and activates ERK, PI3K and phospholipase γ (PLCγ) signalling pathways. Notably, all mutations in PTCs are nearly 100% exclusive. Rearrangements leading to the chimeric protein PAX8-PPARγ were only identified in FTCs (35%) and FTAs (2-10%) [2,3,11]. Additionally, AKT signalling appears most accelerated in ATCs and PTCs due to PTEN mutations and/or PIK3CA amplification [17]. Together the frequency preference of genetic alterations observed in distinct thyroid cancers as well as the observation that well-differentiated thyroid carcinomas precede or co-exist with PDTCs or ATCs supports the view that most undifferentiated thyroid carcinomas evolve by sequential progression (reviewed in: [2]).

In addition to genetic alterations, recent studies indicate that thyroid carcinomas like the majority of as yet analyzed tumors are characterized by aberrant expression of **microRNAs (miRNAs).** These small, non-coding RNAs of 20-24 nucleotides in length are evolutionarily conserved and control gene expression at the post-transcriptional level [18]. At present, 1366 mature human microRNAs are listed in miR-Base (mirbase.org, release 16). The vast majority of identified and characterized microRNAs target the 3’-untranslated region (3’ UTR) of mRNAs and modulate target transcript degradation, translation or both [19,20]. Two classes of microRNAs relevant to cancer are distinguished: **‘onco-miRs’** with tumor promoting effects versus **‘tumor-suppressive’** microRNAs that antagonize cancer progression. In the past years, several studies identified miRNA signatures in thyroid carcinomas aiming to reveal how they modulate thyroid cancer progression and to evaluate their potential for thyroid cancer diagnosis.

## MicroRNA signatures in follicular thyroid carcinomas (FTCs)

Malignant FTCs and benign FTAs share significant similarities at the morphological and molecular level and thus microRNAs could serve as valuable markers to distinguish these tumors. The first study addressing this aspect on the basis of a limited set of human microRNAs (235 distinct human microRNAs) revealed four microRNAs (miR-346, -328, -192, -197) moderately upregulated by 1.34-1.82 fold in FTCs when compared to FTAs [21]. The authors claimed this moderate upregulation sufficed to distinguish FTAs from FTCs in 74% of analyzed patient samples (23 FTCs, 20 FTAs). *In vitro*, the upregulation of miR-197 and -346 was associated with elevated proliferation of HEK293T and two FTC-derived cell lines. This pro-proliferative effect was suggested to correlate with miR-controlled expression of ACVR1 (activin A receptor type 1), TSPAN3 (tetraspanin 3), CFLAR (Caspase 8 and FADD-like apoptosis regulator) and EFEMP2 (fibulin 4), although no direct regulation of these targets by miR-197 or -346 was demonstrated [21]. The abundance of these mRNAs was decreased in the majority of analyzed FTCs and was proposed to distinguish FTAs from FTCs [21]. Notably, previous studies suggested activin signalling to act on growth inhibition of FTC-derived cells *in vitro*, but miRNA-mediated regulation of Activin signalling remains to be shown [22]. TSPAN3 was inversely correlated with the metastatic potential in melanoma and the extracellular matrix (ECM) component EFEMP2 was reported to be over expressed in colon carcinomas but decreased in prostate cancer [23-25].

**Figure 1:**
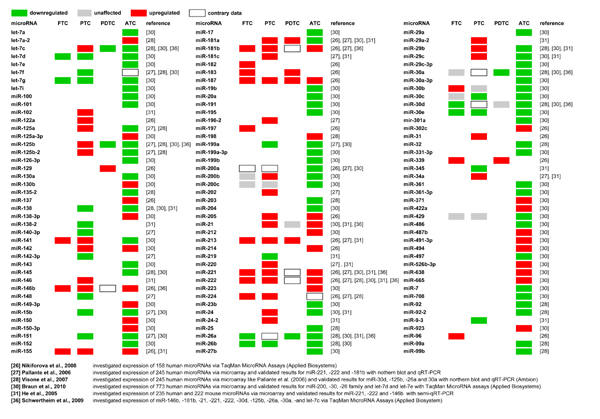
**Deregulated miRNA expression in thyroid carcinomas of follicular origin in comparison to normal thyroid tissue.** Altered expression of miRNAs observed by indicated studies is represented by color coding. Deregulated expression was classified as up- (red), unaffected (grey) or downregulated (green) in comparison to non-transformed thyroid tissue based on individual studies. Contradictory data are indicated by boxes. The presented data base on different thresholds, were quantified using distinct methods and more importantly distinct miRNA sets. Thus, the presented summary of observed miRNA signatures remains incomplete, preliminary and requires substantial further validation. This is particularly important for FTCs and PDTCs for both of which only a very limited set of microRNAs has been analyzed. FTC: Follicular thyroid carcinoma; PTC: Papillary thyroid carcinoma; PDTC: Poorly differentiated thyroid carcinoma, ATC: Anaplastic thyroid carcinoma.

In comprehensive studies aiming at the identification of microRNA signatures distinguishing thyroid carcinomas from non-transformed thyroid tissue (NT), Nikiforova and colleagues [26] could not confirm the results of Weber et al. [21], except for miR-197. This miRNA showed a higher expression in oncocytic FTCs compared to NTs, whereas expression appeared largely unaffected in conventional FTCs [21,26]. Notably, miR-328 expression, previously suggested being higher in FTCs than in FTAs, was found to be upregulated in FTAs but not in FTCs by Nikiforova et al [26]. The increased expression of miR-221/-222 appears to be a hallmark of thyroid cancers of follicular origin, since both miRNAs were found to be expressed at high levels in FTCs, PTCs, PDTCs and ATCs [26-31] (Figure 1). Accordingly, their expression was largely unaffected in FTAs, hyperplastic nodules and medullary thyroid carcinomas (MTCs) [26]. Compared to non-transformed ‘normal’ thyroid (NT) and hyperplastic nodules four additional miRNAs were significantly increased in conventional FTCs (miR-187, -224, -155, -146b) as well as in oncocytic FTCs (miR-187, -339, -183, -197). MiR-187 is one of the ten most upregulated microRNAs in PTCs, FTCs and PDTCs but apparently remains unaffected in FTAs. This suggests miR-187 as a useful marker for distinguishing FTCs from FTAs that however is not suitable for discriminating FTCs from other carcinomas of follicular origin. The only few studies addressing miRNA signatures in FTCs focused exclusively on the upregulation of microRNAs. However, by comparing ATC samples with NTs, FTCs and PTCs, we observed a significant reduction in miR-26a/-b and let-7g expression in FTCs. These miRNAs are considered tumor suppressive miRNAs [32-35].

In conclusion, comprehensive miRNA signature analyses in FTCs, in particular with respect to distinct subtypes like oncocytic versus conventional FTCs, are still lacking. Thus, it remains largely elusive if FTCs can be distinguished from other thyroid carcinomas or FTAs by altered miRNA expression profiles at this point.

## MicroRNA signatures in papillary thyroid carcinomas (PTCs)

The upregulation of the miR-221/-222 cluster and miR-181 is the most consistent finding in all studies addressing miRNA signatures in PTCs [26,27,31,36,37]. Moreover, increased expression of miR-146b, -155, -21 and -220 was reported in at least two individual studies based on comparing signatures between PTCs and NTs (Figure 1). A comparison of PTCs to hyperplastic nodular tissue from formalin fixed paraffin embedded samples further validated the significance of these findings by identifying altered expression of miR-221/-222, -181, -31, and -224 [38]. Upregulation of miR-31 and -224 was also observed by Nikiforova et al. [26]. However, analyses of 40 fine needle aspirate (FNA) specimen and 84 formalin-fixed paraffin-embedded tissues by Chen et al. identified only the upregulation of miR-146b and -222 as potent markers of PTCs [39].

Most interestingly a significant correlation of increased miRNA expression with genetic alterations was observed in PTCs [26]. For instance, miR-221/-222 were more abundant in PTC samples with RAS and BRAF mutations. RAS-mutations moreover correlated with the most severe upregulation of miR-146b. In contrast, RET-mutations were found to be associated with increased expression of miR-155. *In vitro* analyses using thyroid cancer-derived cells confirmed an ‘oncogene connection’ for upregulated expression of miR-221 and -181b. The abundance of these microRNAs increased upon overexpression of v-raf, v-ras, RET/PTC1, RET/PTC3, E1-Abl, E1a-v-raf, middle T of polyomavirus and v-mos [27]. PTC cell lines harboring RET/PTC1 or BRAF V600E mutations identified additional microRNAs to be severely increased. The most severe upregulation upon BRAF mutation was observed for the miR-200 family (miR-200a, -200b, 200c and -141), whereas RET/PTC1 rearrangement was correlated with upregulated expression of miR-128a, -128b, -139 and -200a. The most significantly downregulated group of miRNAs in cells with BRAF mutation comprised miR-127, -130a, and -144; with RET/PTC1 rearrangement miR-154*, -181a, -302b and -302c [40,41].

Although there is substantial and consistent data on the upregulation of microRNAs in PTCs, reduced abundance was only reported for a few miRNAs. The three tumor-suppressive miRNA-families miR-26 [31], -30 [38] and let-7 [27] were suggested to be expressed at lower levels in PTCs. Our recent analyses confirmed these observations for the miR-26 and let-7 families [30]. However, expression of the miR-30 family appeared largely unaffected when compared to NT. In contrast, Schwertheim et al. identified an increased expression of miR-30d, -30a, -26a and let-7c in nine analyzed PTCs compared to NTs [36].

In summary, PTCs appear to be well-distinguishable from NT by altered miRNA expression, in particular the upregulation of onco-miRs. However, more comprehensive analyses including FTCs are required to identify miRNAs expressed at distinct levels in these two carcinoma types.

## MicroRNA signatures in anaplastic thyroid carcinomas (ATCs)

The most striking difference between ATCs and all other thyroid carcinomas of follicular origin appears to be a severely decreased expression of various miRNAs (Figure 1) [28,30,36]. Visone et al. observed decreased expression of 20 and elevated abundance of only four miRNAs (miR-222, -198, -let-7f-1, let-7a-2) by comparing ATCs with non-transformed thyroid tissue [28]. The most significant decrease in expression was determined for miR-30d, -125b-1/2, and -26a which could be confirmed by Schwertheim et al [36]. In support of these findings, we identified 62 down- and 21 upregulated miRNAs compared to non-transformed thyroid [30]. Strikingly, the decreased miRNAs comprised 12 miRNA-families and 12 clustered miRNA transcription units. The most significant downregulation was observed for tumor suppressive miRNAs of the let-7, miR-26, -30 and -200 families. Notably, the comparison of miRNA signatures between ATCs, PTCs and FTCs suggested that downregulation of the miR-30 and -200 families is sufficient to unambiguously distinguish ATCs from NT as well as FTCs and PTCs. As observed for PTCs, the miR-138 was found to be severely reduced in ATC samples as well as in ATC-derived cell lines [30,31,42,43]. Consistent with our studies and the analyses of ATC-derived cells, Nikiforova and colleagues observed a severe upregulation of miR-221/-222 [26,43]. Allthough they examined the expression of 158 microRNAs and identified 57 down- and 47 upregulated in the majority of thyroid tumors they only analyzed the ten most upregulated microRNAs in further detail. Finally, the potent onco-miR-21 was found to be highly expressed in ATCs and was observed to promote thyroid tumor growth in mouse [30,44].

In conclusion, ATCs appear to be well-distinguishable from all other thyroid carcinomas by a severe decrease in the expression of various microRNAs. However, future studies have to reveal if altered miRNA profiles suffice to unambiguously distinguish ATCs from PDTCs.

## MicroRNA signatures in poorly differentiated thyroid carcinomas (PDTCs)

PDTCs are poorly defined and show morphological characteristics of both, differentiated and anaplastic thyroid carcinomas. Aiming to distinguish PDTCs from PTCs and ATCs on the molecular level, Schwertheim et al. investigated the expression of two different sets of microRNAs (‘set 1’: miRNA-146b, -181b, -21, -221 and -222, all upregulated in PTCs; ‘set 2’: miRNA-30d, -125b, -26a, -30a-5p and let7d, all downregulated in ATC). Abundance of these miRNAs was analyzed in comparison to four NTs in 15 PDTCs, nine PTCs and nine ATCs [36]. ‘Set 1’ microRNA expression was slightly increased in PDTCs but did not significantly differ from NTs. Since PTCs and ATCs instead showed a more robust upregulation of these microRNAs, the authors suggested ‘set 1’ microRNAs as a promising diagnostic tool to distinguish PDTCs from PTCs or ATCs. MicroRNAs of ‘set 2’ instead appear useful to discriminate PDTCs from PTCs, since they were expressed at low levels in PDTCs and ATCs but significantly upregulated in PTCs. Nikiforova et al. investigated the microRNA expression of four PDTCs but did not aim to distinguish PDTCs from PTCs or ATCs [26]. In contrast to Schwertheim et al., Nikiforova and colleagues found miR-181b, -221, -222 and -146b to be upregulated in all four analyzed PDTCs when compared to NTs [26].

Although preliminary evidence indicates that miRNA profiles could be useful molecular markers to identify PDTCs, additional and more comprehensive studies are required.

## Onco-miRs in Thyroid cancer

**Figure 2:**
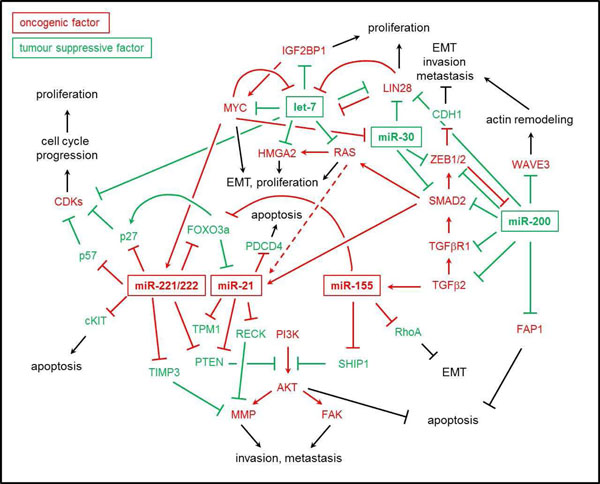
**Regulatory networks facilitated by tumor-suppressive and onco-genic microRNAs / proteins in tumor cells.** Dashed lines indicate indirect regulation.

Onco-miRs are typically classified by an upregulation in cancer and the targeting of transcripts encoding effectors antagonizing tumor progression (Figure 2, red). In the following we discuss validated and suggested phenotypic consequences of aberrant onco-miR expression observed in thyroid cancer.

### miR-221/-222

The overexpression of miR-221/-222 is a hallmark of thyroid malignancies [26-28,30,31]. Moreover, upregulation of both miRNAs was also shown in hepatocellular carcinomas (HCC), glioblastoma and gastric cancer [45-47]. Antagonizing the function of miRs-221/222 in PTC-derived cells interfered with cell proliferation and growth [27]. In subsequent studies it was demonstrated that both miRNAs negatively regulate expression of the cyclin-dependent kinase (CDK) inhibitor p27^Kip1^[29]. This inhibitory role most likely involves miR-221/222 directed control of FOXO3, a key transcriptional activator of p27^Kip1^[48,49]. Notably, FOXO3a mRNA was significantly downregulated in PTCs and FOXO3a represses onco-miR-21 in lung cancer cells [49,50]. An inhibitory role of both miRNAs was also observed for p57^Kip2^ expression [47,51]. Hence, elevated expression of miR-221/-222 presumably indicates aberrant proliferation and cell growth due to their role in antagonizing cell cycle arrest by targeting crucial cell cycle gatekeepers like CDK-inhibitors. Supporting this view, reduced expression of p27^Kip1^ was observed in many primary thyroid tumors as well as thyroid carcinoma-derived cell lines [52].

The expression of the c-kit tyrosine kinase receptor is suppressed or reduced in most tested FTCs and PTCs [53]. Indicating a role of miR-221/-222 in this regulation, it was demonstrated that both miRNAs target c-kit in melanoma-derived cells [54]. Interestingly, 70% of cutaneous melanomas are characterized by BRAF mutations which was also shown to be a precaution of miR-221/-222 upregulation in PTC [26]. Various studies showed that miR-221/-222 downregulate expression of the tumor-suppressive phosphatase PTEN leading to an upregulation of PI3K/AKT signaling [55,56]. This pathway blocks apoptosis and promotes invasion by modulating focal adhesion kinase (FAK) phosphorylation and matrix metalloprotease (MMP) expression levels [57]. PIK3CA amplifications as well as PTEN mutations were reported for many FTCs, PTCs and ATCs suggesting that AKT signaling is sustained by genomic alterations and modified by post-transcriptional control in thyroid carcinomas [17,58,59]. Another pro-apoptotic factor and inhibitor of MMP expression regulated by miR-221/-222 is TIMP3. Reduced expression of PTEN and TIMP3 facilitated by this miRNA-cluster induces TRAIL (tumor necrosis factor (TNF)-related apoptosis inducing ligand) resistance and upregulation of MMPs [55]. *In vivo* evidence for the growth promoting role of elevated miR-221/-222 expression was provided by Xenograft studies in nude mice. The constitutive overexpression of this miRNA cluster resulted in an increased gain of Xenograft weight and volume upon subcutaneous injection of tumor-derived cell lines [47]. Together these findings indicate the overexpression of miR-221/-222 as a key event in thyroid tumor progression. Upregulation of both miRNAs presumably promotes uncontrolled growth and could as well modulate the invasive potential of tumor cell.

### miR-21

Upregulated expression of miR-21 is observed in multiple types of cancers, such as breast, liver, brain, prostate and myometrial cancers but also in PTCs and ATCs [44,60,61]. Most recently it was shown that oncogenic Ras induces miR-21 expression via activating Raf/MAPK and AKT signaling in rat thyroid cells and in a mouse model of lung tumorigenesis [44]. MiR-21 predominantly acts in an anti-apoptotic manner by interfering with the expression of PTEN and PDCD4 [62-64]. Thus, this miRNA promotes AKT signaling similar to the miR-221/-222 cluster and thereby stimulates its own expression. Elevated AKT signaling and the direct regulation of tropomyosin 1 (TPM1) and maspin (PI5) affects cell morphology, motility and enhances cell invasion [65,66]. By the direct targeting of RECK and the indirect effect on TIMP3 expression, upregulation of miR-21 promotes cell migration and invasiveness but also blocks apoptosis [67]. Hence, the overexpression of miR-21 in thyroid cancers is likely to promote uncontrolled growth and potentially upregulated invasiveness of tumor cells.

### miR-155

MiR-155 overexpression was observed in FTCs, PTCs and ATCs [26,31]. In leukemia, miR-155 together with miR-21 stimulates PI3K/AKT signaling by blocking expression of SHIP1, another AKT inhibitory phosphatase [68,69]. In addition miR-155 interferes with the expression of RhoA and serves a role in modulating tumor cell invasion as well as epithelial-to-mesenchymal-transition (EMT) [70]. TGFβ induces miR-155 expression and thereby promotes TGFβ-induced EMT and tight junction dissolution involving the depletion of RhoA. Like miR-221/-222, miR-155 also directly targets FOXO3a [71]. Hence, upregulation of miR-155 in thyroid cancers is presumed to promote cell survival, invasiveness and resistance to chemotherapeutics, as for instance demonstrated in breast cancer cells [71].

## Tumor-suppressive miRNAs in thyroid cancer

In contrast to onco-miRs, tumor-suppressive microRNAs are typically downregulated during tumor progression and target transcripts encoding oncogenic factors. The regulatory role of key tumor-suppressive miRNAs in thyroid cancer is discussed in the following (Figure 2, green).

### miR-200

The miR-200 family (comprising miR-141, -200a, -200b, - 200c and -429) was identified as a potent suppressor of EMT [72-75]. Reduced expression of this miRNA-family was previously reported for stomach, breast and ovarian cancers [76]. Surprisingly, moderately upregulated expression of some miR-200 family members was observed in PTCs and FTCs, whereas a severe downregulation of this microRNA family was found in all ATCs analyzed [30]. This observation supports the view that the miR-200 family serves a key role in preserving an epithelial phenotype or morphology, respectively. Elevated levels of the miR-200 family interfere with the expression of EMT-promoting factors like ZEB1, ZEB2, SNAI2, SMAD2, TGFβR1 and TGFβ2. This antagonizes transcriptional repression of E-cadherin and the miR-200 genomic clusters (miR-141/-200c and miR-200a/-200b/-429) [30,72-74]. In agreement, severely reduced E-cadherin levels have been described as a common characteristic of primary ATCs as well as ATC-derived cells [30,77]. Moreover, elevated expression of TGFβ2 was observed in ATC and PDTC compared to FTC and PTC samples [78]. Beyond the antagonistic role in TGFβ-induced EMT, the miR-200 family presumably also modulates actin dynamics by interfering with the expression of WAVE3, an actin cytoskeleton remodeling protein [79]. Taken together, this provides strong evidence that the loss of miR-200 expression presents a hallmark in the progression of thyroid carcinomas culminating in TGFβ-dependent EMT and elevated invasiveness, as observed for ATCs [30]. The loss of miR-200 expression is probably potentiated by TGFβ-mediated upregulation of miR-155 expression and enhancement of miR-21-maturation, for both of which a pro-metastatic function was proposed [70,80]. More recently, regulation of LIN28B by miR-200 members was identified in prostate cancer-derived cells [81]. LIN28 is a stem cell factor and a powerful inducer of pluripotency [82]. It also represses maturation of the tumor-suppressive let-7 family by binding to the loop region of let-7 precursors [83-85]. Additionally, the miR-200 family apparently promotes apoptosis, as demonstrated in colorectal cancer-derived HCT116 cells. In these cells the repression of FAP1 (Fas-associated phosphatase 1) by the miR-200 family was correlated with elevated susceptibility to TNF-receptor CD95-dependent apoptosis [86]. In summary, these findings indicate reduced expression of the miR-200 family as a key trigger for dedifferentiation and potentiated aggressiveness observed in ATCs.

### let-7

Downregulation of the let-7 family is observed in all thyroid carcinomas of follicular origin [27,28,30]. Let-7 was initially identified as a factor promoting differentiation in C. elegans and was since then validated as a key regulator of gene expression in various organisms [87,88]. The let-7 family is ubiquitously expressed in adult mouse tissue and its reduced expression is considered to be a hallmark in cancer progression [89]. In accord with their tumor-suppressive role, members of the let-7 family target cell cycle regulators and oncogenes like RAS, HMGA1/2, MYC, IGF2BP1 and LIN28 [34,89-93]. Thus it appears likely that the capability to antagonize activating RAS mutations and thus uncontrolled proliferation is severely compromised by reduced levels of the let-7 family. Like activating RAS mutations, the overexpression of MYC is associated with enhanced cell growth and reduced serum dependency in various malignancies [94]. Accordingly, the frequently observed increase of MYC levels in ATCs is likely to correlate with a downregulation of the let-7 family [78,95,96]. Notably, MYC is a key regulator of miRNA transcription and negatively controls expression of various tumor-suppressive miRNA clusters including once again the let-7 family but in addition the miR-30, -26, -34 and -29 families [97,98]. Another layer of post-transcriptional regulation is provided by the control of MYC mRNA degradation via the RNA binding protein IGF2BP1 (Insulin-like growth factor 2 mRNA binding protein 1) [99]. IGF2BP1 shows an oncofetal expression pattern and becomes *de novo* synthesized in various cancers [100]. This is in agreement with a reduction of let-7 expression, since members of this miRNA family interfere with IGF2BP1 expression. Thus, MYC-directed repression of the let-7 family is likely to promote the expression of IGF2BP1 that in turn sustains MYC expression [97]. HMGA1 and HMGA2, two other key factors in tumor progression that are controlled by the let-7 family, support the RAS/MEK-facilitated induction of EMT by enhancing SNAIL expression [101-103]. Moreover, HMGA proteins promote cell growth and HMGA1 depletion induces programmed cells death in ATC-derived cells [104]. In conclusion, loss of the let-7 family is likely to be an early event in the progression of thyroid carcinomas. The sever reduction of this tumor-suppressive miRNA family promotes uncontrolled tumor growth and presumably also affects the invasive potential of tumor cells at later stages.

### miR-30

Significantly reduced expression of the miR-30 family was identified in ATCs. However, moderately reduced expression is also observed in FTCs, PTCs and PDTCs, with the exception of miR-30b in FTCs [28,30]. The first hint of a potential tumor-suppressive role of this miRNA family was revealed by the identification of UBC9 as a direct target [105]. Downregulation of this E2-conjugating enzyme for sumoylation interferes with cell growth and cancer progression. In bladder cancer, reduced expression of the miR-30 family was associated with upregulation of the tumor marker cytokeratin 7, although a functional role of this potential regulation remains elusive [106]. We identified the miR-30 family as an antagonist of TGFβ-induced EMT and *in vitro* invasiveness of ATC-derived cells [30]. This role apparently involves the direct targeting of SMAD2 and ZEB2. In agreement, the forced expression of miR-30 family members interfered with *in vitro* invasiveness of ATC-derived cells and correlated with a downregulation of the mesenchymal marker vimentin. However, in contrast to the miR-200 family, expression of the miR-30 family is apparently controlled in a ZEB-independent manner [30]. Intriguingly, the miR-30 family also targets the stem cell factor LIN28, as previously demonstrated for other key tumor-suppressive miRNA families like miR-200 and let-7 [107]. Hence, the identification of deregulated expression of the miR-30 family in ATCs identified yet another tumor-suppressive miRNA cluster modulating EMT and invasiveness of tumor cells.

## Conclusions

The comprehensive view of distinct miRNA signatures in thyroid carcinomas of follicular origin provides novel insights in the molecular pathologies of these malignancies. However, knowledge of target mRNAs controlled by deregulated miRNAs in thyroid cancers is still sparse at present. To reveal how altered expression of microRNAs promotes or antagonizes thyroid tumor progression it is thus required to identify novel miRNA targets in future studies.

In thyroid carcinomas the most striking observation is that ‘less’ aggressive FTCs and PTCs (compared to ATCs) are apparently characterized by an upregulation of ‘oncogenic’ miRNAs (e.g. miR-221/-222 or miR-146b). These miRNAs mainly act in a pro-proliferative and anti-apoptotic manner. In accord with the clonal progression hypothesis, ATCs reveal a severe reduction of tumor-suppressive miRNAs (Figure 1). This decrease presumably promotes dedifferentiation, which morphologically manifests as an epithelial-to-mesenchymal-transition (EMT) driven by reduced expression of the miR-200 and -30 families (see Fig. 1 in [30].). Notably, reduction of these miRNA families is likely to provide a valuable diagnostic tool for distinguishing ATCs from FTC or PTCs. Unresolved aspects of immediate diagnostic and potentially therapeutic importance address the question if miRNA signatures are suitable to distinguish FTAs from FTCs. The presented studies seem to be insufficient to define a subset of microRNAs that unambiguously discriminates these thyroid cancers. Thus, it is required to re-evaluate miRNA signatures in thyroid cancers of follicular origin in a comprehensive manner based on pathologically unambiguously classified primary samples. These studies need to be based on the same evaluation method, for instance miRNA-microarrays, to generate data sets allowing an unbiased direct comparison of miRNA signatures.

Despite their potential as diagnostic tools, miRNAs could also provide promising therapeutic targets based on onco-miR inhibition or restoring levels of tumor suppressive miRNAs. The stage for such approaches was set in rodents already in 2005 by demonstrating that the role of miR-16 as well as -122 could be antagonized by the intravenous injection of anti-miRs [108]. Vice versa, the adenoviral delivery of the tumor-suppressive miR-26 was shown to suppress mouse liver tumorigenesis [33]. However, in clinical practice the specificity of miRNA or anti-miR delivery remains an essential limitation due to off-site effects in non-neoplastic organs.

A third strategy could be envisioned on the level of epigenetic silencing of tumor-suppressive miRNAs. In breast and prostate cancer cell lines methylation of the miR-200 cluster was observed and inhibition of methyl-transferases released this transcription block [109]. Moreover, epigenetic silencing might explain the observation that complete miRNA transcription units are downregulated in ATCs (Table1 in [30]). Hence, spatially restricted and cluster specific release of epigenetic silencing could provide a valuable tool in the treatment of cancer but as yet such approaches are not available.

In conclusion the here reviewed findings reveal the potency and current limitations of miRNAs in diagnosis, prognosis and potentially therapeutic strategies for the treatment of thyroid cancer. However, substantial additional work will be required to establish miRNAs in clinical practice and reveal the molecular networks via which altered miRNA expression promotes thyroid cancer progression.

## Competing interests

The authors declare that they have no competing interests.

## Authors’ contribution

Both authors contributed equally

## References

[B1] Jemal A Bray F Center MM Ferlay J Ward E Forman D Global cancer statistics CA Cancer J Clin 61 2 69 90 10.3322/caac.20107 21296855

[B2] Kondo T Ezzat S Asa SL Pathogenetic mechanisms in thyroid follicular-cell neoplasia Nat Rev Cancer 2006 6 292 306 10.1038/nrc1836 16557281

[B3] Schmid KW Molecular pathology of thyroid tumors Pathologe 2010 31 Suppl 2 229 233 20717681 10.1007/s00292-010-1321-2

[B4] Williams ED Guest Editorial: Two Proposals Regarding the Terminology of Thyroid Tumors Int J Surg Pathol 2000 8 181 183 10.1177/106689690000800304 11493987

[B5] DeLellis RA L RV Heitz PU Eng C World Health Organization Classification of Tumours, Pathology and Genetics of Tumours of Endocrine Organs 2004 49 133

[B6] Ain KB Anaplastic thyroid carcinoma: a therapeutic challenge Semin Surg Oncol 1999 16 64 69 10.1002/(SICI)1098-2388(199901/02)16:1<64::AID-SSU10>3.0.CO;2-U 9890741

[B7] Pilotti S Collini P Mariani L Placucci M Bongarzone I Vigneri P Cipriani S Falcetta F Miceli R Pierotti MA Rilke F Insular carcinoma: a distinct de novo entity among follicular carcinomas of the thyroid gland Am J Surg Pathol 1997 21 1466 1473 10.1097/00000478-199712000-00009 9414190

[B8] Williams D Cancer after nuclear fallout: lessons from the Chernobyl accident Nat Rev Cancer 2002 2 543 549 10.1038/nrc845 12094241

[B9] Harach HR Escalante DA Day ES Thyroid cancer and thyroiditis in Salta, Argentina: a 40-yr study in relation to iodine prophylaxis Endocr Pathol 2002 13 175 181 10.1385/EP:13:3:175 12446916

[B10] Gudmundsson J Sulem P Gudbjartsson DF Jonasson JG Sigurdsson A Bergthorsson JT He H Blondal T Geller F Jakobsdottir M Common variants on 9q22.33 and 14q13.3 predispose to thyroid cancer in European populations Nat Genet 2009 41 460 464 10.1038/ng.339 19198613 PMC3664837

[B11] Nikiforova MN Nikiforov YE Molecular diagnostics and predictors in thyroid cancer Thyroid 2009 19 1351 1361 10.1089/thy.2009.0240 19895341

[B12] Pallante P Visone R Croce CM Fusco A Deregulation of microRNA expression in follicular-cell-derived human thyroid carcinomas Endocr Relat Cancer 2010 17 F91 104 10.1677/ERC-09-0217 19942715

[B13] Wan PT Garnett MJ Roe SM Lee S Niculescu-Duvaz D Good VM Jones CM Marshall CJ Springer CJ Barford D Marais R Mechanism of activation of the RAF-ERK signaling pathway by oncogenic mutations of B-RAF Cell 2004 116 855 867 10.1016/S0092-8674(04)00215-6 15035987

[B14] Knauf JA Ma X Smith EP Zhang L Mitsutake N Liao XH Refetoff S Nikiforov YE Fagin JA Targeted expression of BRAFV600E in thyroid cells of transgenic mice results in papillary thyroid cancers that undergo dedifferentiation Cancer Res 2005 65 4238 4245 10.1158/0008-5472.CAN-05-0047 15899815

[B15] Garcia-Rostan G Camp RL Herrero A Carcangiu ML Rimm DL Tallini G Beta-catenin dysregulation in thyroid neoplasms: down-regulation, aberrant nuclear expression, and CTNNB1 exon 3 mutations are markers for aggressive tumor phenotypes and poor prognosis Am J Pathol 2001 158 987 996 10.1016/S0002-9440(10)64045-X 11238046 PMC1850336

[B16] Miyake N Maeta H Horie S Kitamura Y Nanba E Kobayashi K Terada T Absence of mutations in the beta-catenin and adenomatous polyposis coli genes in papillary and follicular thyroid carcinomas Pathol Int 2001 51 680 685 10.1046/j.1440-1827.2001.01269.x 11696170

[B17] Paes JE Ringel MD Dysregulation of the phosphatidylinositol 3-kinase pathway in thyroid neoplasia Endocrinol Metab Clin North Am 2008 37 375 387 viii-ix 10.1016/j.ecl.2008.01.001 18502332 PMC2446602

[B18] Ghildiyal M Zamore PD Small silencing RNAs: an expanding universe Nat Rev Genet 2009 10 94 108 10.1038/nrg2504 19148191 PMC2724769

[B19] Fabian MR Sonenberg N Filipowicz W Regulation of mRNA translation and stability by microRNAs Annu Rev Biochem 2010 79 351 379 10.1146/annurev-biochem-060308-103103 20533884

[B20] Rigoutsos I New tricks for animal microRNAS: targeting of amino acid coding regions at conserved and nonconserved sites Cancer Res 2009 69 3245 3248 10.1158/0008-5472.CAN-09-0352 19351814

[B21] Weber F Teresi RE Broelsch CE Frilling A Eng C A limited set of human MicroRNA is deregulated in follicular thyroid carcinoma J Clin Endocrinol Metab 2006 91 3584 3591 10.1210/jc.2006-0693 16822819

[B22] Schulte KM Jonas C Krebs R Roher HD Activin A and activin receptors in thyroid cancer Thyroid 2001 11 3 14 10.1089/10507250150500603 11272093

[B23] Boucheix C Duc GH Jasmin C Rubinstein E Tetraspanins and malignancy Expert Rev Mol Med 2001 2001 1 17 14987371 10.1017/S1462399401002381

[B24] Gallagher WM Greene LM Ryan MP Sierra V Berger A Laurent-Puig P Conseiller E Human fibulin-4: analysis of its biosynthetic processing and mRNA expression in normal and tumour tissues FEBS Lett 2001 489 59 66 10.1016/S0014-5793(00)02389-9 11231014

[B25] Wlazlinski A Engers R Hoffmann MJ Hader C Jung V Muller M Schulz WA Downregulation of several fibulin genes in prostate cancer Prostate 2007 67 1770 1780 10.1002/pros.20667 17929269

[B26] Nikiforova MN Tseng GC Steward D Diorio D Nikiforov YE MicroRNA expression profiling of thyroid tumors: biological significance and diagnostic utility J Clin Endocrinol Metab 2008 93 1600 1608 10.1210/jc.2007-2696 18270258 PMC2386678

[B27] Pallante P Visone R Ferracin M Ferraro A Berlingieri MT Troncone G Chiappetta G Liu CG Santoro M Negrini M MicroRNA deregulation in human thyroid papillary carcinomas Endocr Relat Cancer 2006 13 497 508 10.1677/erc.1.01209 16728577

[B28] Visone R Pallante P Vecchione A Cirombella R Ferracin M Ferraro A Volinia S Coluzzi S Leone V Borbone E Specific microRNAs are downregulated in human thyroid anaplastic carcinomas Oncogene 2007 26 7590 7595 10.1038/sj.onc.1210564 17563749

[B29] Visone R Russo L Pallante P De Martino I Ferraro A Leone V Borbone E Petrocca F Alder H Croce CM Fusco A MicroRNAs (miR)-221 and miR-222, both overexpressed in human thyroid papillary carcinomas, regulate p27Kip1 protein levels and cell cycle Endocr Relat Cancer 2007 14 791 798 10.1677/ERC-07-0129 17914108

[B30] Braun J Hoang-Vu C Dralle H Huttelmaier S Downregulation of microRNAs directs the EMT and invasive potential of anaplastic thyroid carcinomas Oncogene 2010 29 4237 4244 10.1038/onc.2010.169 20498632

[B31] He H Jazdzewski K Li W Liyanarachchi S Nagy R Volinia S Calin GA Liu CG Franssila K Suster S The role of microRNA genes in papillary thyroid carcinoma Proc Natl Acad Sci U S A 2005 102 19075 19080 10.1073/pnas.0509603102 16365291 PMC1323209

[B32] Ji J Shi J Budhu A Yu Z Forgues M Roessler S Ambs S Chen Y Meltzer PS Croce CM MicroRNA expression, survival, and response to interferon in liver cancer N Engl J Med 2009 361 1437 1447 10.1056/NEJMoa0901282 19812400 PMC2786938

[B33] Kota J Chivukula RR O'Donnell KA Wentzel EA Montgomery CL Hwang HW Chang TC Vivekanandan P Torbenson M Clark KR Therapeutic microRNA delivery suppresses tumorigenesis in a murine liver cancer model Cell 2009 137 1005 1017 10.1016/j.cell.2009.04.021 19524505 PMC2722880

[B34] Boyerinas B Park SM Shomron N Hedegaard MM Vinther J Andersen JS Feig C Xu J Burge CB Peter ME Identification of let-7-regulated oncofetal genes Cancer Res 2008 68 2587 2591 10.1158/0008-5472.CAN-08-0264 18413726

[B35] Ricarte-Filho JC Fuziwara CS Yamashita AS Rezende E da-Silva MJ Kimura ET Effects of let-7 microRNA on Cell Growth and Differentiation of Papillary Thyroid Cancer Transl Oncol 2009 2 236 241 19956384 10.1593/tlo.09151 PMC2781070

[B36] Schwertheim S Sheu SY Worm K Grabellus F Schmid KW Analysis of deregulated miRNAs is helpful to distinguish poorly differentiated thyroid carcinoma from papillary thyroid carcinoma Horm Metab Res 2009 41 475 481 10.1055/s-0029-1215593 19370508

[B37] Sheu SY Grabellus F Schwertheim S Worm K Broecker-Preuss M Schmid KW Differential miRNA expression profiles in variants of papillary thyroid carcinoma and encapsulated follicular thyroid tumours Br J Cancer 2010 102 376 382 10.1038/sj.bjc.6605493 20029416 PMC2816660

[B38] Tetzlaff MT Liu A Xu X Master SR Baldwin DA Tobias JW Livolsi VA Baloch ZW Differential expression of miRNAs in papillary thyroid carcinoma compared to multinodular goiter using formalin fixed paraffin embedded tissues Endocr Pathol 2007 18 163 173 10.1007/s12022-007-0023-7 18058265

[B39] Chen YT Kitabayashi N Zhou XK Fahey TJ 3rd Scognamiglio T MicroRNA analysis as a potential diagnostic tool for papillary thyroid carcinoma Mod Pathol 2008 21 1139 1146 10.1038/modpathol.2008.105 18587330

[B40] Cahill S Smyth P Denning K Flavin R Li J Potratz A Guenther SM Henfrey R O'Leary JJ Sheils O Effect of BRAFV600E mutation on transcription and post-transcriptional regulation in a papillary thyroid carcinoma model Mol Cancer 2007 6 21 10.1186/1476-4598-6-21 17355635 PMC1831483

[B41] Cahill S Smyth P Finn SP Denning K Flavin R O'Regan EM Li J Potratz A Guenther SM Henfrey R Effect of ret/PTC 1 rearrangement on transcription and post-transcriptional regulation in a papillary thyroid carcinoma model Mol Cancer 2006 5 70 10.1186/1476-4598-5-70 17156473 PMC1713250

[B42] Takakura S Mitsutake N Nakashima M Namba H Saenko VA Rogounovitch TI Nakazawa Y Hayashi T Ohtsuru A Yamashita S Oncogenic role of miR-17-92 cluster in anaplastic thyroid cancer cells Cancer Sci 2008 99 1147 1154 10.1111/j.1349-7006.2008.00800.x 18429962 PMC11160010

[B43] Mitomo S Maesawa C Ogasawara S Iwaya T Shibazaki M Yashima-Abo A Kotani K Oikawa H Sakurai E Izutsu N Downregulation of miR-138 is associated with overexpression of human telomerase reverse transcriptase protein in human anaplastic thyroid carcinoma cell lines Cancer Sci 2008 99 280 286 10.1111/j.1349-7006.2007.00666.x 18201269 PMC11159409

[B44] Frezzetti D Menna MD Zoppoli P Guerra C Ferraro A Bello AM Luca PD Calabrese C Fusco A Ceccarelli M Upregulation of miR-21 by Ras in vivo and its role in tumor growth Oncogene 30 275 286 10.1038/onc.2010.416 20956945

[B45] Pineau P Volinia S McJunkin K Marchio A Battiston C Terris B Mazzaferro V Lowe SW Croce CM Dejean A miR-221 overexpression contributes to liver tumorigenesis Proc Natl Acad Sci U S A 2010 107 264 269 10.1073/pnas.0907904107 20018759 PMC2806773

[B46] Ciafre SA Galardi S Mangiola A Ferracin M Liu CG Sabatino G Negrini M Maira G Croce CM Farace MG Extensive modulation of a set of microRNAs in primary glioblastoma Biochem Biophys Res Commun 2005 334 1351 1358 10.1016/j.bbrc.2005.07.030 16039986

[B47] Kim YK Yu J Han TS Park SY Namkoong B Kim DH Hur K Yoo MW Lee HJ Yang HK Kim VN Functional links between clustered microRNAs: suppression of cell-cycle inhibitors by microRNA clusters in gastric cancer Nucleic Acids Res 2009 37 1672 1681 10.1093/nar/gkp002 19153141 PMC2655672

[B48] Di Leva G Gasparini P Piovan C Ngankeu A Garofalo M Taccioli C Iorio MV Li M Volinia S Alder H MicroRNA cluster 221-222 and estrogen receptor alpha interactions in breast cancer J Natl Cancer Inst 2010 102 706 721 10.1093/jnci/djq102 20388878 PMC2873185

[B49] Karger S Weidinger C Krause K Sheu SY Aigner T Gimm O Schmid KW Dralle H Fuhrer D FOXO3a: a novel player in thyroid carcinogenesis? Endocr Relat Cancer 2009 16 189 199 18845647 10.1677/ERC-07-0283

[B50] Wang K Li PF Foxo3a regulates apoptosis by negatively targeting miR-21 J Biol Chem 2010 285 16958 16966 10.1074/jbc.M109.093005 20371612 PMC2878079

[B51] Fornari F Gramantieri L Ferracin M Veronese A Sabbioni S Calin GA Grazi GL Giovannini C Croce CM Bolondi L Negrini M MiR-221 controls CDKN1C/p57 and CDKN1B/p27 expression in human hepatocellular carcinoma Oncogene 2008 27 5651 5661 10.1038/onc.2008.178 18521080

[B52] Baldassarre G Belletti B Bruni P Boccia A Trapasso F Pentimalli F Barone MV Chiappetta G Vento MT Spiezia S Overexpressed cyclin D3 contributes to retaining the growth inhibitor p27 in the cytoplasm of thyroid tumor cells J Clin Invest 1999 104 865 874 10.1172/JCI6443 10510327 PMC408550

[B53] Natali PG Berlingieri MT Nicotra MR Fusco A Santoro E Bigotti A Vecchio G Transformation of thyroid epithelium is associated with loss of c-kit receptor Cancer Res 1995 55 1787 1791 7536131

[B54] Igoucheva O Alexeev V MicroRNA-dependent regulation of cKit in cutaneous melanoma Biochem Biophys Res Commun 2009 379 790 794 10.1016/j.bbrc.2008.12.152 19126397

[B55] Garofalo M Di Leva G Romano G Nuovo G Suh SS Ngankeu A Taccioli C Pichiorri F Alder H Secchiero P miR-221&222 regulate TRAIL resistance and enhance tumorigenicity through PTEN and TIMP3 downregulation Cancer Cell 2009 16 498 509 10.1016/j.ccr.2009.10.014 19962668 PMC2796583

[B56] Chun-Zhi Z Lei H An-Ling Z Yan-Chao F Xiao Y Guang-Xiu W Zhi-Fan J Pei-Yu P Qing-Yu Z Chun-Sheng K MicroRNA-221 and microRNA-222 regulate gastric carcinoma cell proliferation and radioresistance by targeting PTEN BMC Cancer 2010 10 367 10.1186/1471-2407-10-367 20618998 PMC2914702

[B57] Brader S Eccles SA Phosphoinositide 3-kinase signalling pathways in tumor progression, invasion and angiogenesis Tumori 2004 90 2 8 15143962 10.1177/030089160409000102

[B58] Hou P Ji M Xing M Association of PTEN gene methylation with genetic alterations in the phosphatidylinositol 3-kinase/AKT signaling pathway in thyroid tumors Cancer 2008 113 2440 2447 10.1002/cncr.23869 18831514

[B59] Hou P Liu D Shan Y Hu S Studeman K Condouris S Wang Y Trink A El-Naggar AK Tallini G Genetic alterations and their relationship in the phosphatidylinositol 3-kinase/Akt pathway in thyroid cancer Clin Cancer Res 2007 13 1161 1170 10.1158/1078-0432.CCR-06-1125 17317825

[B60] Jazbutyte V Thum T MicroRNA-21: from cancer to cardiovascular disease Curr Drug Targets 2010 11 926 935 10.2174/138945010791591403 20415649

[B61] Cho WC OncomiRs: the discovery and progress of microRNAs in cancers Mol Cancer 2007 6 60 10.1186/1476-4598-6-60 17894887 PMC2098778

[B62] Meng F Henson R Lang M Wehbe H Maheshwari S Mendell JT Jiang J Schmittgen TD Patel T Involvement of human micro-RNA in growth and response to chemotherapy in human cholangiocarcinoma cell lines Gastroenterology 2006 130 2113 2129 10.1053/j.gastro.2006.02.057 16762633

[B63] Frankel LB Christoffersen NR Jacobsen A Lindow M Krogh A Lund AH Programmed cell death 4 (PDCD4) is an important functional target of the microRNA miR-21 in breast cancer cells J Biol Chem 2008 283 1026 1033 17991735 10.1074/jbc.M707224200

[B64] Lu Z Liu M Stribinskis V Klinge CM Ramos KS Colburn NH Li Y MicroRNA-21 promotes cell transformation by targeting the programmed cell death 4 gene Oncogene 2008 27 4373 4379 10.1038/onc.2008.72 18372920 PMC11968769

[B65] Asangani IA Rasheed SA Nikolova DA Leupold JH Colburn NH Post S Allgayer H MicroRNA-21 (miR-21) post-transcriptionally downregulates tumor suppressor Pdcd4 and stimulates invasion, intravasation and metastasis in colorectal cancer Oncogene 2008 27 2128 2136 10.1038/sj.onc.1210856 17968323

[B66] Zhu S Wu H Wu F Nie D Sheng S Mo YY MicroRNA-21 targets tumor suppressor genes in invasion and metastasis Cell Res 2008 18 350 359 10.1038/cr.2008.24 18270520

[B67] Gabriely G Wurdinger T Kesari S Esau CC Burchard J Linsley PS Krichevsky AM MicroRNA 21 promotes glioma invasion by targeting matrix metalloproteinase regulators Mol Cell Biol 2008 28 5369 5380 10.1128/MCB.00479-08 18591254 PMC2519720

[B68] Yamanaka Y Tagawa H Takahashi N Watanabe A Guo YM Iwamoto K Yamashita J Saitoh H Kameoka Y Shimizu N Aberrant overexpression of microRNAs activate AKT signaling via down-regulation of tumor suppressors in natural killer-cell lymphoma/leukemia Blood 2009 114 3265 3275 10.1182/blood-2009-06-222794 19641183

[B69] Pedersen IM Otero D Kao E Miletic AV Hother C Ralfkiaer E Rickert RC Gronbaek K David M Onco-miR-155 targets SHIP1 to promote TNFalpha-dependent growth of B cell lymphomas EMBO Mol Med 2009 1 288 295 10.1002/emmm.200900028 19890474 PMC2771872

[B70] Kong W Yang H He L Zhao JJ Coppola D Dalton WS Cheng JQ MicroRNA-155 is regulated by the transforming growth factor beta/Smad pathway and contributes to epithelial cell plasticity by targeting RhoA Mol Cell Biol 2008 28 6773 6784 10.1128/MCB.00941-08 18794355 PMC2573297

[B71] Kong W He L Coppola M Guo J Esposito NN Coppola D Cheng JQ MicroRNA-155 regulates cell survival, growth, and chemosensitivity by targeting FOXO3a in breast cancer J Biol Chem 2010 285 17869 17879 10.1074/jbc.M110.101055 20371610 PMC2878550

[B72] Gregory PA Bert AG Paterson EL Barry SC Tsykin A Farshid G Vadas MA Khew-Goodall Y Goodall GJ The miR-200 family and miR-205 regulate epithelial to mesenchymal transition by targeting ZEB1 and SIP1 Nat Cell Biol 2008 10 593 601 10.1038/ncb1722 18376396

[B73] Park SM Gaur AB Lengyel E Peter ME The miR-200 family determines the epithelial phenotype of cancer cells by targeting the E-cadherin repressors ZEB1 and ZEB2 Genes Dev 2008 22 894 907 10.1101/gad.1640608 18381893 PMC2279201

[B74] Burk U Schubert J Wellner U Schmalhofer O Vincan E Spaderna S Brabletz T A reciprocal repression between ZEB1 and members of the miR-200 family promotes EMT and invasion in cancer cells EMBO Rep 2008 9 582 589 10.1038/embor.2008.74 18483486 PMC2396950

[B75] Korpal M Lee ES Hu G Kang Y The miR-200 family inhibits epithelial-mesenchymal transition and cancer cell migration by direct targeting of E-cadherin transcriptional repressors ZEB1 and ZEB2 J Biol Chem 2008 283 14910 14914 10.1074/jbc.C800074200 18411277 PMC3258899

[B76] Brabletz S Brabletz T The ZEB/miR-200 feedback loop--a motor of cellular plasticity in development and cancer? EMBO Rep 2010 11 670 677 10.1038/embor.2010.117 20706219 PMC2933868

[B77] Smallridge RC Marlow LA Copland JA Anaplastic thyroid cancer: molecular pathogenesis and emerging therapies Endocr Relat Cancer 2009 16 17 44 18987168 10.1677/ERC-08-0154 PMC2829440

[B78] Montero-Conde C Martin-Campos JM Lerma E Gimenez G Martinez-Guitarte JL Combalia N Montaner D Matias-Guiu X Dopazo J de Leiva A Molecular profiling related to poor prognosis in thyroid carcinoma. Combining gene expression data and biological information Oncogene 2008 27 1554 1561 10.1038/sj.onc.1210792 17873908

[B79] Sossey-Alaoui K Bialkowska K Plow EF The miR200 family of microRNAs regulates WAVE3-dependent cancer cell invasion J Biol Chem 2009 284 33019 33029 10.1074/jbc.M109.034553 19801681 PMC2785142

[B80] Davis BN Hilyard AC Lagna G Hata A SMAD proteins control DROSHA-mediated microRNA maturation Nature 2008 454 56 61 10.1038/nature07086 18548003 PMC2653422

[B81] Kong D Banerjee S Ahmad A Li Y Wang Z Sethi S Sarkar FH Epithelial to mesenchymal transition is mechanistically linked with stem cell signatures in prostate cancer cells PLoS One 2010 5 e12445 10.1371/journal.pone.0012445 20805998 PMC2929211

[B82] Yu J Vodyanik MA Smuga-Otto K Antosiewicz-Bourget J Frane JL Tian S Nie J Jonsdottir GA Ruotti V Stewart R Induced pluripotent stem cell lines derived from human somatic cells Science 2007 318 1917 1920 10.1126/science.1151526 18029452

[B83] Newman MA Thomson JM Hammond SM Lin-28 interaction with the Let-7 precursor loop mediates regulated microRNA processing RNA 2008 14 1539 1549 10.1261/rna.1155108 18566191 PMC2491462

[B84] Viswanathan SR Daley GQ Gregory RI Selective blockade of microRNA processing by Lin28 Science 2008 320 97 100 10.1126/science.1154040 18292307 PMC3368499

[B85] Rybak A Fuchs H Smirnova L Brandt C Pohl EE Nitsch R Wulczyn FG A feedback loop comprising lin-28 and let-7 controls pre-let-7 maturation during neural stem-cell commitment Nat Cell Biol 2008 10 987 993 10.1038/ncb1759 18604195

[B86] Schickel R Park SM Murmann AE Peter ME miR-200c regulates induction of apoptosis through CD95 by targeting FAP-1 Mol Cell 2010 38 908 915 10.1016/j.molcel.2010.05.018 20620960 PMC2904349

[B87] Reinhart BJ Slack FJ Basson M Pasquinelli AE Bettinger JC Rougvie AE Horvitz HR Ruvkun G The 21-nucleotide let-7 RNA regulates developmental timing in Caenorhabditis elegans Nature 2000 403 901 906 10.1038/35002607 10706289

[B88] Pasquinelli AE Reinhart BJ Slack F Martindale MQ Kuroda MI Maller B Hayward DC Ball EE Degnan B Muller P Conservation of the sequence and temporal expression of let-7 heterochronic regulatory RNA Nature 2000 408 86 89 10.1038/35040556 11081512

[B89] Johnson CD Esquela-Kerscher A Stefani G Byrom M Kelnar K Ovcharenko D Wilson M Wang X Shelton J Shingara J The let-7 microRNA represses cell proliferation pathways in human cells Cancer Res 2007 67 7713 7722 10.1158/0008-5472.CAN-07-1083 17699775

[B90] Johnson SM Grosshans H Shingara J Byrom M Jarvis R Cheng A Labourier E Reinert KL Brown D Slack FJ RAS is regulated by the let-7 microRNA family Cell 2005 120 635 647 10.1016/j.cell.2005.01.014 15766527

[B91] Sampson VB Rong NH Han J Yang Q Aris V Soteropoulos P Petrelli NJ Dunn SP Krueger LJ MicroRNA let-7a down-regulates MYC and reverts MYC-induced growth in Burkitt lymphoma cells Cancer Res 2007 67 9762 9770 10.1158/0008-5472.CAN-07-2462 17942906

[B92] Lee YS Dutta A The tumor suppressor microRNA let-7 represses the HMGA2 oncogene Genes Dev 2007 21 1025 1030 10.1101/gad.1540407 17437991 PMC1855228

[B93] Guo Y Chen Y Ito H Watanabe A Ge X Kodama T Aburatani H Identification and characterization of lin-28 homolog B (LIN28B) in human hepatocellular carcinoma Gene 2006 384 51 61 16971064 10.1016/j.gene.2006.07.011

[B94] Armelin HA Armelin MC Kelly K Stewart T Leder P Cochran BH Stiles CD Functional role for c-myc in mitogenic response to platelet-derived growth factor Nature 1984 310 655 660 10.1038/310655a0 6088986

[B95] Kurihara T Ikeda S Ishizaki Y Fujimori M Tokumoto N Hirata Y Ozaki S Okajima M Sugino K Asahara T Immunohistochemical and sequencing analyses of the Wnt signaling components in Japanese anaplastic thyroid cancers Thyroid 2004 14 1020 1029 10.1089/thy.2004.14.1020 15650354

[B96] Chang TC Zeitels LR Hwang HW Chivukula RR Wentzel EA Dews M Jung J Gao P Dang CV Beer MA Lin-28B transactivation is necessary for Myc-mediated let-7 repression and proliferation Proc Natl Acad Sci U S A 2009 106 3384 3389 10.1073/pnas.0808300106 19211792 PMC2651245

[B97] Chang TC Yu D Lee YS Wentzel EA Arking DE West KM Dang CV Thomas-Tikhonenko A Mendell JT Widespread microRNA repression by Myc contributes to tumorigenesis Nat Genet 2008 40 43 50 10.1038/ng.2007.30 18066065 PMC2628762

[B98] Mott JL Kurita S Cazanave SC Bronk SF Werneburg NW Fernandez-Zapico ME Transcriptional suppression of mir-29b-1/mir-29a promoter by c-Myc, hedgehog, and NF-kappaB J Cell Biochem 2010 110 1155 1164 10.1002/jcb.22630 20564213 PMC2922950

[B99] Prokipcak RD Herrick DJ Ross J Purification and properties of a protein that binds to the C-terminal coding region of human c-myc mRNA J Biol Chem 1994 269 9261 9269 8132663

[B100] Yisraeli JK VICKZ proteins: a multi-talented family of regulatory RNA-binding proteins Biol Cell 2005 97 87 96 15601260 10.1042/BC20040151

[B101] Watanabe S Ueda Y Akaboshi S Hino Y Sekita Y Nakao M HMGA2 maintains oncogenic RAS-induced epithelial-mesenchymal transition in human pancreatic cancer cells Am J Pathol 2009 174 854 868 10.2353/ajpath.2009.080523 19179606 PMC2665746

[B102] Thuault S Tan EJ Peinado H Cano A Heldin CH Moustakas A HMGA2 and Smads co-regulate SNAIL1 expression during induction of epithelial-to-mesenchymal transition J Biol Chem 2008 283 33437 33446 10.1074/jbc.M802016200 18832382 PMC2662269

[B103] Selbach M Schwanhausser B Thierfelder N Fang Z Khanin R Rajewsky N Widespread changes in protein synthesis induced by microRNAs Nature 2008 455 58 63 10.1038/nature07228 18668040

[B104] Scala S Portella G Fedele M Chiappetta G Fusco A Adenovirus-mediated suppression of HMGI(Y) protein synthesis as potential therapy of human malignant neoplasias Proc Natl Acad Sci U S A 2000 97 4256 4261 10.1073/pnas.070029997 10759549 PMC18219

[B105] Wu F Zhu S Ding Y Beck WT Mo YY MicroRNA-mediated regulation of Ubc9 expression in cancer cells Clin Cancer Res 2009 15 1550 1557 10.1158/1078-0432.CCR-08-0820 19223510 PMC2846614

[B106] Ichimi T Enokida H Okuno Y Kunimoto R Chiyomaru T Kawamoto K Kawahara K Toki K Kawakami K Nishiyama K Identification of novel microRNA targets based on microRNA signatures in bladder cancer Int J Cancer 2009 125 345-352 10.1002/ijc.24390 19378336

[B107] Zhong X Li N Liang S Huang Q Coukos G Zhang L Identification of microRNAs regulating reprogramming factor LIN28 in embryonic stem cells and cancer cells J Biol Chem 2010 10.1074/jbc.M110.169607 PMC3009922 20947512

[B108] Krutzfeldt J Rajewsky N Braich R Rajeev KG Tuschl T Manoharan M Stoffel M Silencing of microRNAs in vivo with 'antagomirs' Nature 2005 438 685 689 10.1038/nature04303 16258535

[B109] Vrba L Jensen TJ Garbe JC Heimark RL Cress AE Dickinson S Stampfer MR Futscher BW Role for DNA methylation in the regulation of miR-200c and miR-141 expression in normal and cancer cells PLoS One 2010 5 e8697 10.1371/journal.pone.0008697 20084174 PMC2805718

